# Expression of renal cell markers and detection of 3p loss links endolymphatic sac tumor to renal cell carcinoma and warrants careful evaluation to avoid diagnostic pitfalls

**DOI:** 10.1186/s40478-018-0607-0

**Published:** 2018-10-19

**Authors:** Rachel Jester, Iya Znoyko, Maria Garnovskaya, Joseph N Rozier, Ryan Kegl, Sunil Patel, Tuan Tran, Malak Abedalthagafi, Craig M Horbinski, Mary Richardson, Daynna J Wolff, Razvan Lapadat, William Moore, Fausto J Rodriguez, Jason Mull, Adriana Olar

**Affiliations:** 1 Department of Pathology and Laboratory Medicine, Medical University of South Carolina, 171 Ashley Ave, Charleston, 29425 SC USA; 2 Department of Neurosurgery, Medical University of South Carolina, 171 Ashley Ave, Charleston, 29425 SC USA; 3 Department of Pathology, Baylor University Medical Center, 3500 Gaston Ave, Dallas, 75246 TX USA; 4 Genomics Research Department, Saudi Humane Genome Project, King Fahad Medical City and King Abdulaziz City for Science and Technology, Riyadh, Saudi Arabia; 5 Department of Pathology and Neurosurgery, Feinberg School of Medicine, Northwestern University, 251 E. Huron St, Chicago, 60611 IL USA; 6 Department of Pathology, Loyola University Medical Center, 2160 S 1st Ave, Maywood, 60153 IL USA; 7 Department of Radiology, UT Southwestern Medical Center, 5323 Harry Hines Blvd, Dallas, 75390 TX USA; 8 Department of Pathology, Johns Hopkins Hospital, 1800 Orleans St, Baltimore, 21287 MD USA; 9 Department of Pathology, UT Southwestern Medical Center, 5323 Harry Hines Blvd, Dallas, 75390 TX USA; 10 Hollings Cancer Center, 86 Jonathan Lucas Street, Charleston, 29425 SC USA

**Keywords:** Endolymphatic sac tumor, Renal cell carcinoma, VHL, PAX-8, PAX-2, CA-9, Copy number profiles

## Abstract

Endolymphatic sac tumor (ELST) is a rare neoplasm arising in the temporal petrous region thought to originate from endolymphatic sac epithelium. It may arise sporadically or in association with Von-Hippel-Lindau syndrome (VHL). The ELST prevalence in VHL ranges from 3 to 16% and may be the initial presentation of the disease. Onset is usually in the 3rd to 5th decade with hearing loss and an indolent course. ELSTs present as locally destructive lesions with characteristic computed tomography imaging features. Histologically, they show papillary, cystic or glandular architectures. Immunohistochemically, they express keratin, EMA, and variably S100 and GFAP. Currently it is recommended that, given its rarity, ELST needs to be differentiated from other entities with similar morphologic patterns, particularly other VHL-associated neoplasms such as metastatic clear cell renal cell carcinoma (ccRCC). Nineteen ELST cases were studied. Immunohistochemistry (18/19) and single nucleotide polymorphism microarray testing was performed (12/19). Comparison with the immunophenotype and copy number profile in RCC is discussed. Patients presented with characteristic bone destructive lesions in the petrous temporal bones. Pathology of tumors showed characteristic ELST morphology with immunoexpression of CK7, GFAP, S100, PAX-8, PAX-2, CA-9 in the tumor cells. Immunostaines for RCC, CD10, CK20, chromogranin A, synaptophysin, TTF-1, thyroglobulin, and transthyretin were negative in the tumor cells. Molecular testing showed loss of 3p and 9q in 66% (8/12) and 58% (7/12) cases, respectively. Immunoreactivity for renal markers in ELST is an important diagnostic caveat and has not been previously reported. In fact, renal markers are currently recommended in order to rule out metastatic RCC although PAX gene complex and CA-9 have been implicated in the development of the inner ear. Importantly copy number assessment of ELST has not been previously reported. Loss of 3p (including the *VHL* locus) in ELST suggests similar mechanistic origins as ccRCC.

**Electronic supplementary material:**

The online version of this article (10.1186/s40478-018-0607-0) contains supplementary material, which is available to authorized users.

## Introduction

Von-Hippel Lindau (VHL) is an autosomal dominant hereditary cancer predisposition syndrome characterized by abberations in the *VHL* tumor supressor gene at chromosome location 3p25.3. Patients with VHL are at increased risk of developing a variety of neoplasasms such as central nervous system (CNS) hemangioblastomas, clear cell renal cell carcinomas (ccRCC), pheochromocytomas and extra-adrenal paragangliomas, pancreatic neuroendocrine tumors and adenomas, and endolymphatic sac tumors (ELST) of the inner ear [7, 32].

ELST are very rare tumors of neuroectodermal origin, thought to arise from the rugose, intraosseous portion of the endolymphatic sac. The endolymphatic sac represents an extension of the membranous labyrinth that follows the endolymphatic duct. It has both intraosseous and extraosseous components and ends in a blind pouch in the dura mater lining the posterior surface of the temporal bone. Histologically, the endolymphatic sac is composed of a single layer of flat to cuboidal to low and tall columnar cells resting on a basement membrane with folds and papillae formation in the inferomedial aspect of the intraosseous portion [1, 3, 18]. ELSTs more commonly arise sporadically, but also may arise in association with VHL, with a prevalence of up to approximately 16%. ELST typically occur around the 3rd to 5th decade and present with sensorineural hearing loss, tinnitus, and cranial nerve palsies on the affected side [2, 7]. Radiographically on computed tomography (CT), the tumors are characterized by heterogenous bone destruction centered on the posterior portion of the temporal bone. On magnetic resonance imaging (MRI), ELST demostrate T1 post-contrast enhancement and bright T2 signal [3, 7]. Grossly, tumors are described as blue to red in color and hypervascular [7, 37]. The histologic appearance of ELST ranges from a follicular growth pattern with colloid-filled cystic spaces to a papillary arrangement with solid and hypercellular areas, and occasionally an epithelioid clear cell pattern. The neoplastic cells are arranged in a single cuboidal layer with uniform nuclei and minimal pleomorphism, mitotic activity, or necrosis. Tumors are cytokeratin positive and immunoexpress epithelial membrane antigen (EMA), vimentin, neuron specific enolase (NSE), glialfibrillary acidic protein (GFAP), and variable S100, vascular endothelial growth factor (VEGF), and synaptophysin [14, 39].

The radiographic and histologic appearance of ELST raises a broad differential diagnosis and one must be aware of other more common conditions that may mimic such in order to avoid diagnostic pitfalls [18]. In fact, ELSTs are often not diagnosed until after the initial interpretation is questioned clinically [3]. Entities to consider in the work-up of suspected ELST include choroid plexus tumors, paragaganglioma, and metastatic papillary thyroid carcinoma, tumors which would be expected to immunoexpress transthyretin, chromogranin/synapthophysin, and TTF-1/thyroglobulin, respectively [7, 39]. Of particular importance to rule out are other VHL-associated neoplasms such as metastatic ccRCC. Immunohistochemistry for paired box (PAX) transcription factors PAX-8 and PAX-2, carbonic anhydrase 9 (CA-9), RCC, and CD10 has been suggested for this purpose and are reportedly negative in ELST [7, 25, 32, 39].

In this paper we characterize a cohort of ELST and demostrate immunoreactivity for renal cell markers as well as molecular evidence of predominant 3p and 9q loss which has not been previously described. Loss of 3p (including the *VHL* locus) in ELST suggests similar mechanistic origins as ccRCC. These findings are important, both to correct the previous assumption that renal cell immunohistochemical markers should not be expressed by ELST, which is important for diagnosis, and also to further characterize this rare neoplasm in order to better understand its pathogenesis.

## Materials and Methods

Cases of ELST were identified via search of the laboratory information system and details regarding patient demographics, presentation, and imaging were collected through electronic medical record review with the approval of the institutional review board from all institutions. Hematoxylin and eosin (H&E) stained slides were reviewed and the diagnosis confirmed. The best tumor block was selected for DNA extraction. All immunohistochemical and molecular testing was performed on formalin-fixed, paraffin embedded (FFPE) tissues except in one case where only fresh frozen tumor tissue was available.

### Immunohistochemistry

Antibodies were validated according to protocol with appropriate tissue controls. Four μm sections were prepared for immunohistochemical evaluation with the following antibodies (clone, dilution, antigen retrieval, supplier): CK7 (OV-TL 12/30, 1:500, citrate, Cell Marque, Rocklin, California, USA), CK20 (Ks20.8, 1:500, EDTA, Cell Marque, Rocklin, CA, USA), PAX-8 (MRQ-50, 1:3000, EDTA, Cell Marque, Rocklin, CA, USA), RCC (PN-15, 1:500, protease, Cell Marque, Rocklin, CA, USA), CD10 (56C6, 1:1000, EDTA, Cell Marque, Rocklin, CA, USA), CA-9 (MRQ-54, 1:2000, EDTA, Cell Marque, Rocklin, CA, USA), GFAP (EP672Y, 1:200, EDTA, Cell Marque, Rocklin, CA, USA), thyroglobulin (2H11+6E1, 1:5000, EDTA, Cell Marque, Rocklin, CA, USA), S100 (4C4.9, 1:4000, EDTA, Cell Marque, Rocklin, CA, USA), chromogranin A (LK2H10, 1:6000, citrate, Cell Marque, Rocklin, CA, USA), synaptophysin (MRQ-40, 1:5000, citrate, Cell Marque, Rocklin, CA, USA), PAX-2 (EP235, 1:1000, citrate, Cell Marque, Rocklin, CA, USA), transthyretin (rabbit polyclonal, 1:15000, citrate, Boster Biological Technology, Pleasanton, CA, USA), TTF-1 (EP229, 1:500, EDTA, Cell Marque, Rocklin, CA, USA), and Ki-67 (SP6, 1:500, EDTA, Cell Marque, Rocklin, CA, USA). Visualization was performed using the HiDef Detection™ HRP Polymer System (Cell Marque, Rocklin, CA, USA) with diaminobenzidine substrate (Cell Marque, Rocklin, CA, USA) and with hematoxylin counterstain in order to visualize the antibody-antigen complex and background tissue, respectively.

### Single nucleotide polymorphism (SNP)-microarray

Genomic DNA extraction for SNP-microarray analysis was performed using the Maxwell® CSC DNA FFPE Kit (Promega, Madison, WI, USA) as detailed by the manufacturer. Microarray-based chromosome analysis of copy number and genotype data was performed according to the manufacturer’s protocol using the IScan System with the Infinium CytoSNP-850 K v1.1 BeadChip (Illumina, Inc., San Diego, CA, USA) and analyzed using GenomeStudio (Illumina, Inc), and Nexus, version 9.0 (BioDiscovery, Inc., El Segundo, CA, USA) software. The signal intensity was determined using the log_2_ ratio and was used along with the specific allele (B-allele) frequency to evaluate copy number patterns of aberrations (clonal changes in less than 100% of cells including deletion, duplication, loss of heterozygosity, ploidy) and genotype, and assessed visually using the KaryoStudio and Nexus files, by comparing with standard curve data charts generated by computer modeling of mosaicism (SiDCoN [simulated DNA copy number]) [26]. Non-mosaic aberrations (those found in 100% of cells) were not included in data analysis as they were considered to represent constitutional changes.

### Statistical methods and bioinformatics

Descriptive statistics were performed in Microsoft® Excel® 2013 (15.0.4971.1000), 64-bit. Gene mapping and visualization was performed using the hg19 assembly, UCSC Genome Browser tool suite [17, 19]. Pathway analysis and protein interactions was performed using Ingenuity Pathway Analysis (IPA) v. 01–07 (Qiagen Inc., https://www.qiagenbioinformatics.com/products/ingenuity-pathway-analysis/) [20].

## Results

### Clinical data

Nineteen ESLT from 10 males and 9 females were analyzed. The median age at diagnosis was 45.1 years (mean: 42.8, range: 14.7–63.3). One patient had known VHL disease confirmed by sequencing (*VHL* exon 2 heterozygous deletion identified) with multiple small nodular cerebellar lesions on imaging suggestive of hemangioblastomas (biopsy not performed). None of the patients had clinical history of RCC. Abdominal imaging when available (9/19) showed no evidence of a renal mass. Patients presented with symptoms including hearing loss, balance difficulties, and unilateral facial paralysis.

### Imaging

**Fig. 1:**
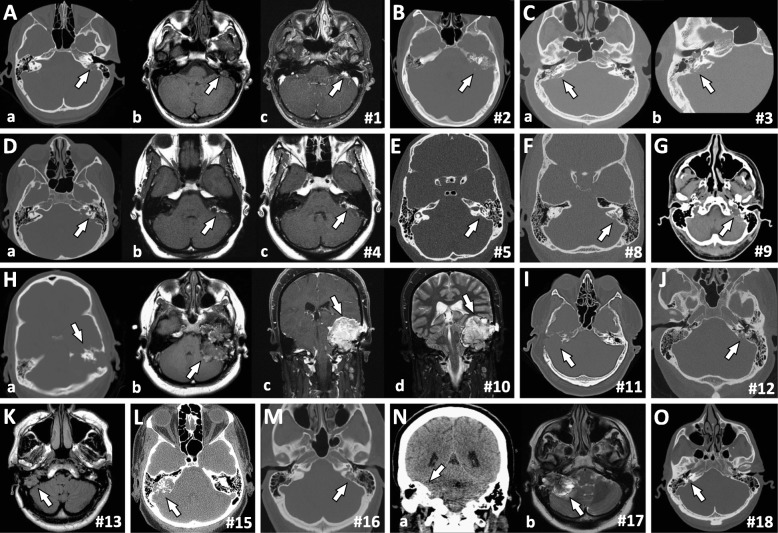
Imaging Findings: Computed tomography shows bone destructive lesions involving the posterior aspect of the petrous temporal bones (**Aa, B, C, Da, E-G, Ha, I, J, L, M, Na, O**). Magnetic resonance imaging shows expansile complex partially solid and cystic masses centered on the petrous segment of the temporal bones demonstrating heterogeneous intrinsic T1 (**Ab, Db, Hb**) and T2 signal (**Hd, Nb**) and post-contrast enhancement (**Ac, Dc, Hc**). (# represents case number). Note: All images are pre-operative except K which is an axial T1-weighted post-operative (at recurrence) image

On CT, all patients showed unilateral expansile bone-destructive lesions involving the posterior petrous segment of the temporal bones. Tumors reached up to 6.4 cm in largest dimension (Case 10). Larger lesions were complex in nature with combined solid and cystic patterns (Fig. 1, Case 10 and 17) and mass effect in the posterior cranial fossa (Case 17). On MRI the lesions showed heterogeneous T1 and T2 signal and post-contrast enhancement on T1-weighted sequences (Fig. 1, Cases 1, 4, 10, 17). Some tumors demonstrated regions of intrinsic T1 hyperintensity, which has been described and is thought to correlate with internal hemorrhage or proteinaceous material (Fig. 1Ab, Db, Hb) [6, 23].

### Gross features

Tumors were tan-pink to red in color and many were described intraoperatively as “vascular” appearing. Some were pulsating intraoperatively, and were initially thought to represent glomus jugulare tumors.

### Microscopy

**Fig. 2:**
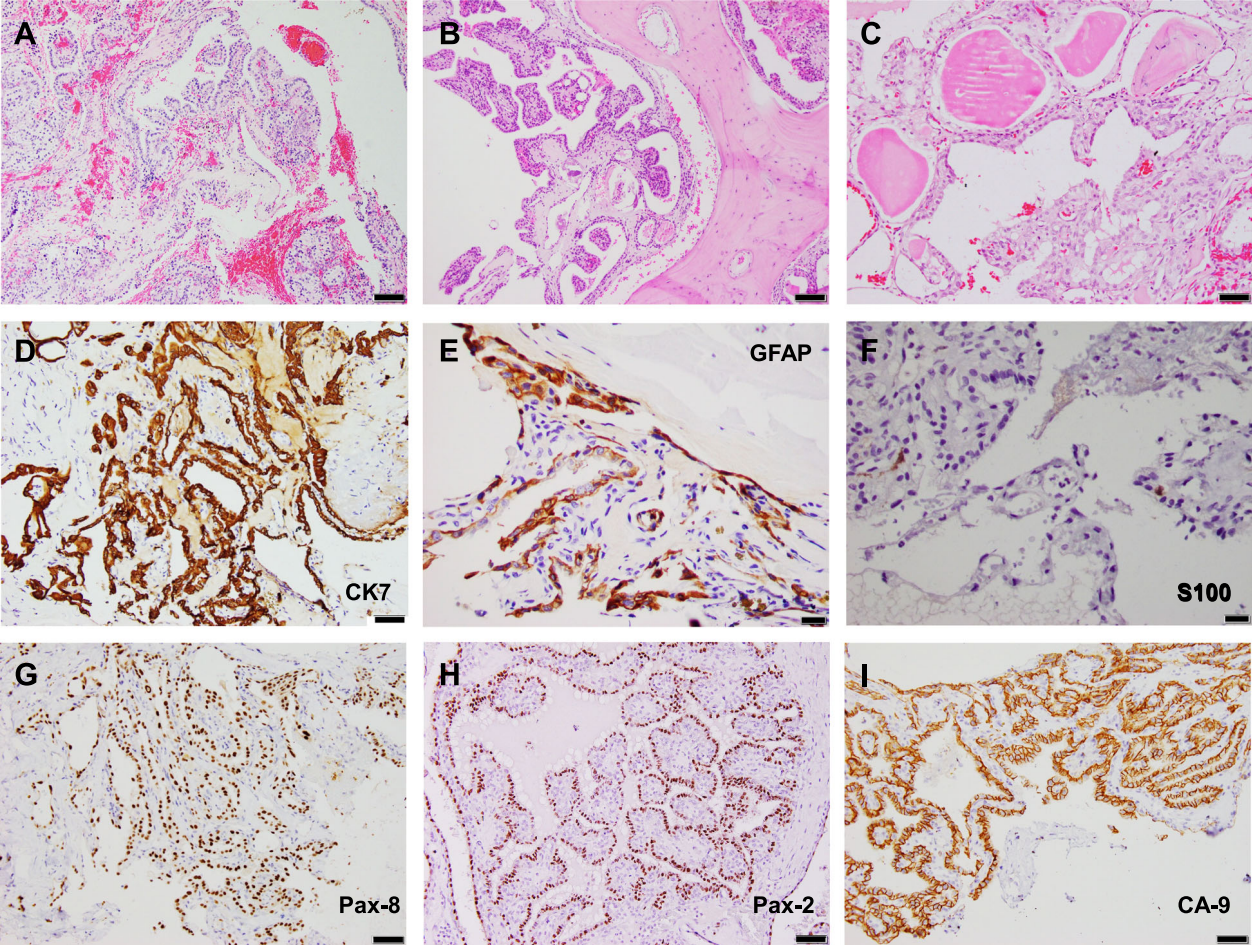
Histological and immunophenotypical findings: All tumors showed the characteristic papillary architecture (**a, b, 100X**) with bone invasion (**b**) and some showed follicular morphology (**c, 200X**). All tumors immunoexpressed GFAP (**d, 400X**), CK7 (**e, 200X**), PAX-8 (**f, 200X**), PAX-2 (**g, 200X**), and CA-9 (**h, 200X**). S100 was focally immunoexpressed (**i, 400X**) in all but one case

**Fig. 3:**
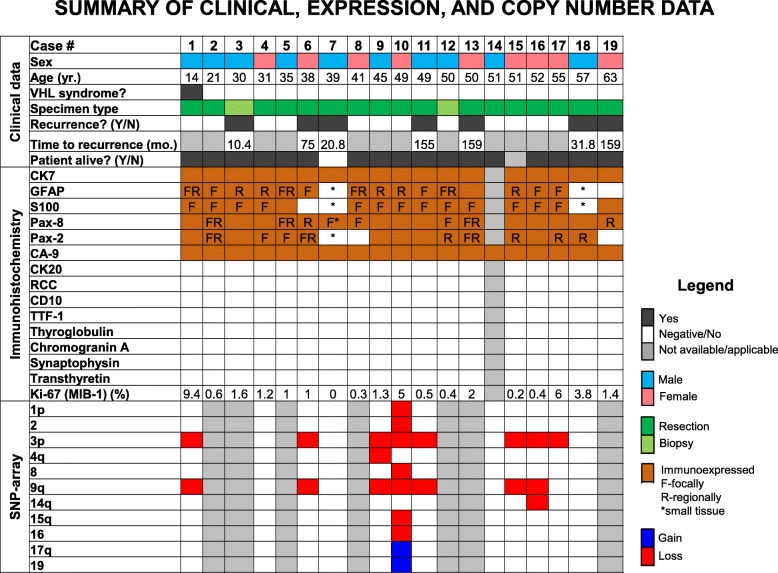
Overview of the clinical, immunohistochemical, and molecular results. Abbreviations: mo.-months; N-no; Y-yes; yr.-years

The histopathological appearance of ELST displayed characteristic papillary architecture with a single-cell epithelial lining and central fibrovascular cores. The surface epithelial cells were cuboidal to cylindrical, bland in appearance, with vacuolated to clear cytoplasm and round to elongated nuclei. Several cases showed cystic growth with glandular spaces filled with an eosinophilic colloid-like material (Fig. 2). Immunohistochemically, the neoplastic cells showed diffuse expression of CK7 (18/18, 100%), CA-9 (19/19, 100%), focal to regional GFAP (15/18, 83.33%), PAX-8 (18/18, 100%), PAX-2 (15/18, 83.33%), and S100 (15/18, 83.33%). The tumor cells were negative for CK20, synaptophysin, chromogranin A, transthyretin, TTF-1, RCC, thyroglobulin, and CD10. Ki-67 (MIB-1) showed a < 10% proliferative index for all cases (1000 tumor nuclei counted) (median: 1.1, mean: 1.97, range: 0–9.4) (Figs. 2 and 3).

### Copy number alterations

**Fig. 4:**
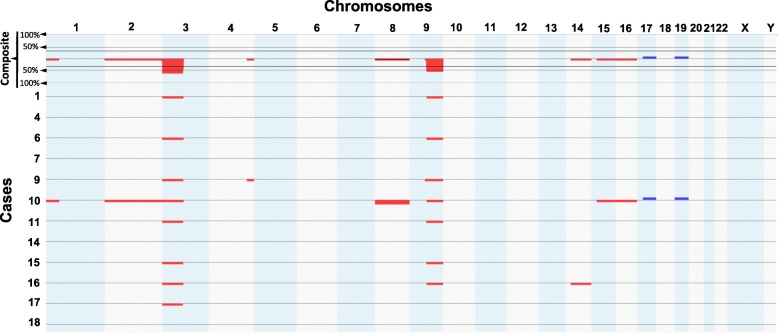
Overview of the SNP-microarray findings: Loss of 3p in 8/12 cases and loss of 9q in 7/12 cases, copy number changes present in clear cell renal cell carcinoma. Legend: chromosomal gains are depicted in blue, losses are depicted in red

Of 12 cases tested by SNP-microarray, 8 demonstrated loss of 3p of which 7 also showed loss of 9q. A detailed summary of the chromosomal regions involved in presented in Figs. 3, 4 and Table 1. Four cases had no abnormalities detected on SNP-microarray.

**Table 1:** SNP-microarray results

Case #	Summary	ISCN 2016
1	-3p, −9q	arr[GRCh37] 3p26.3p11.1(1_90450511)×1[0.3],9q21.11q34.3(70715485_141213431)×1[0.3]
4		arr(1–22,X)x2 normal female
6	-3p, −9q	arr[GRCh37] 3p26.3q11.1(1_91025539)×1[0.2],9q21.11q34.3(70726185_141213431)×1[0.2]
7		arr(1–22)x2,(X,Y)× 1 normal male
9	-3p, −4q, −9q	arr[GRCh37] 3p26.3p11.1(1_89605910)×1[0.1],4q32.1q35.2(160067846_191154276)×1[0.1],9q12q34.3(63455393_141011985)×1[0.1]
10	-1p, −2, −3p, −4q, −8, −9q, −14q, −15q, −16, −17q, − 19	arr[GRCh37] 1p36.33p32.2(1_56794840)×1[0.2],2p25.3-q37.3(1_243199373)×1[0.2],3p26.3p11.1(1_91000000)×1[0.5],8p23.3-q24.3(1_146364022)×0[0.2],9q21.11q34.3(70965125_141213431)×1[0.5],15q11.2-q26.3(22437778_102531392)×1[0.2],16p13.3-q24.3(1_90354753)×1[0.2],17q11.1-q25.3(25295032_81195210)×3[0.2],19p13.3-q13.43(0_59128983)×3[0.2]
11	-3p, −9q	arr[GRCh37] 3p26.3p11.1(1_89317847)×1[0.5],9q21.11q34.3(69901656_141213431)×1[0.5]
14		arr(1–22)x2,(X,Y)×1 normal male
15	-3p, −9q	arr[GRCh37] 3p26.3p11.1(1_89189701)×1[0.2],9q21.11q34.3(70251958_141213431)×1[0.2]
16	-3p, −9q, −14q	arr[GRCh37] 3p26.3p11.1(1_90311584)×1[0.2],9q21.11q34.3(70618596_141213431)×1[0.2],14q11.2q32.33(19754766_107349540)×1[0.2]
17	-3p	arr[GRCh37] 3p26.3p11.1(1_88135518)×1[0.15]
18		arr(1–22)x2,(X,Y)×1 normal male

*Abbreviations*: *ISCN* the International System for Human Cytogenomic Nomenclature

### Pathway and protein interaction analyses

Using 584 and 686 genes mapped on 3p and 9q chromosomal regions, respectively, we investigated which of the encoded proteins potentially interact with PAX-2, PAX-8, CA-9, HIF-1, and GFAP. Relevant genes on chromosome 3p were: *CTNNB1*, *CAND2*, *VHL*, *MIFT*, *WNT7A*, *PDCD6IP*, *TGFBR2*, *PRKCD*, and *MST1*. Relevant genes on chromosome 9q were: *LMX1B*, *GOLGA2*, *HSPA5*, *LCN2*, *RAD23B*, *TLR4*, *KLF4*, and *NOTCH1.* The protein products of these genes are involved in cancer activation pathways like WNT, mTOR, HIF-1alpha, renal cell carcinoma, and p53 signaling, regulation of epithelial to mesenchymal transition, and neuroinflammation signaling (Additional file 1). Based on interactions with PAX-2, PAX-8, and CA-9 the main candidates for ELST tumorigenesis were *VHL* (on 3p), *KLF4* (on 9q), and *CTNNB1* (*beta-catenin*) (on 3p).

### Treatment and follow-up

Treatment involved surgical resection of the mass in all except 2 cases (3 and 12) that underwent biopsy only (Fig. 3). None of the patients with complete medical records (15/19) received chemotherapy. Two patients received radiation therapy. One patient (case 9) received gamma knife radiotherapy to what was considered to be an intraoperative macroscopic glomus jugulare tumor (tissue was not sent to pathology), 8 and 5 years before a histologic diagnosis of ELST was rendered. Another patient (case 11) received radiation at initial diagnosis and at recurrence. Seven patients experienced recurrence of the ELST (Fig. 3), 4 of which required additional surgery (cases 6, 7, 13, and 19), and three of which underwent radiation therapy (cases 6, 11, and 18). One of the patients who experienced tumor recurrence passed away (case 7); however, it is not known with certainty whether this was related to disease or not. One patient was lost to follow-up after his initial resection (case 15) (Fig. 3).

## Discussion

In this paper we present a cohort of patients with ELSTs and show that the majority of tumors immunoexpress renal cell markers. This novel finding is an important diagnostic caveat for general surgical pathology practice and we emphasize the importance of correlating the clinical pathological and radiological findings before rendering a final diagnosis for tumors involving the posterior temporal bone. In addition, we demonstrate combined loss of 3p (including the *VHL* locus) and 9q in the majority (58%, 7/12) of the tested cases by SNP-microarray analysis. These novel molecular findings suggest similar mechanistic origins between sporadic ELST and ccRCC with *VHL* likely playing a central role.

Immunoexpression of CA-9, PAX-8, and PAX-2 prove an important diagnostic caveat when attempting to rule out metastatic RCC in the work-up of suspected ELST. While these markers are more commonly associated with RCC, there are a number of syndromes that link the kidney to the inner ear suggesting certain embryological and functional similarities between the two organ systems [12, 36]. Torban et al. investigated the parallel functions between these two organ systems and categorized diseases affecting both the kidney and ear into groups that A) arose from mutations in shared developmental genes [e.g. Branchio-Oto-renal (BOT) syndrome, Hypoparathyroidism, Deafness, and Renal Dysplasia (HDR) syndrome, Townes-Brocks syndrome (TBS), Kallmann syndrome], B) involved defective ciliary function (e.g. Bardet-Biedl syndrome–associated hearing loss, Alstrom syndrome, nephronophthisis-associated inner ear dysfunction) and C) were due to disruption of specialized transport or structural proteins [e.g. Distal Renal Tubular Acidosis with Deafness (dRTA), Alport syndrome, Bartter syndrome with deafness] [36].

Paired box genes encode a family of transcription factors with roles in organogenesis [12]. PAX-8 activation is tightly linked to PAX-2 (Additional file 1) and these proteins are essential for proper ear [4] and kidney development [27]. In particular, *PAX-2* has been shown to play a role in the induction of inner ear development and commitment of progenitor cells to the formation of the otic placode, the earliest structure identified in the morphogenesis of the inner ear, calling into question its anecdotic specificity for the kidney in the surgical pathology community [9]. PAX-2 is highly expressed in proliferating areas of the early developing inner ear (otic placode and otic vesicle) and is downregulated in areas of apoptosis at later stages of development and in maturing, differentiated hair cells [21, 22]. Its important role in cochlear development has been demonstrated using knockout mice [4, 5]. PAX-8 is one of the earliest markers for the ectodermally-derived otic placode and intermediate mesoderm, having a central role in auditory and urinary system development [12, 27]. It seems that in the neoplastic cells of ELSTs PAX-2 and PAX-8 become upregulated, reproducing the proliferative stages of early development. We speculate the role of an upstream activation mechanism which possibly involves VHL-KLF4-CTNNB1 protein interactions (Additional file 1). *VHL* is a tumor suppressor with known roles in tumorigenesis [7, 32]. VHL physically interacts with Krüppel-like factor 4 (KLF4), a transcription factor with roles in tumorigenesis [8]. Human KLF4 induces increased mouse *PAX-2* and *CTNNB1* mRNA expression [31] and, importantly, KLF4 binds to CTNNB1 inhibiting WNT signaling [13, 34]. In addition, it has been shown that gain-of-function CTNNB1 mutant protein promotes increased expression of *PAX-8* mRNA in mice [28].

The enzyme carbonic anhydrase is distributed in a wide variety of organ systems, including the renal tubules and the inner ear. Within the ear, carbonic anhydrase has activity in the cochlear hair cells, supporting cells surrounding the sensory hair cells in the vestibule, in the stria vascularis, and in the epithelial cells of the rugose portion of the endolymphatic sac. It is thought to play a role in regulating the pH and ionic balance of the endolymph [15]. CA-9 is upregulated in hypoxic conditions and has been shown to play a role in tumorigenesis by altering pH to promote tumor growth and survival in a number of tumors, notably renal cell carcinoma [24, 29]. CA-9 expression is regulated by VHL. Briefly, under normal conditions VHL binds HIF-1alpha and degrades it, preventing its binding to HIF-1beta. Mutant VHL or normal VHL under hypoxic conditions facilitates HIF-1alpha binding to HIF1-beta and HIF-1 protein complex formation which causes downstream transcription of hypoxia-inducible genes such as CA-9. CA-9 is a marker of hypoxia [16, 29, 30, 32, 33]. CA-9 immunoexpression in ELST along with 3p loss provides supporting evidence of *VHL* deficiency in the mechanism of ELST development.

Furthermore, both syndromic and sporadic ELSTs have been shown to harbor mutations in the *VHL* gene [35, 38]; however, to the best of our knowledge molecular profiling of ELST has not yet been attempted [3, 18]. Our finding of 3p loss in ELST links it to ccRCC, which is characterized by variably sized deletions in the short arm of chromosome 3, including the *VHL* tumor suppressor gene [10, 11, 29]. Loss of 9q is also frequently seen in ccRCC, where it, along with 14q deletions, denote a poorer prognosis [10]. In our cohort, only one ELST patient had combined 3p/9q/14q losses but no tumor recurrence after 1.6 years of follow-up (Case 16). While 3p deletions in ccRCC may encompass the entire chromosome arm or only a small portion around the *VHL* locus, the 8 ELST cases in our cohort harboring 3p alterations showed whole p arm losses. Moreover, the 7 cases with concurrent 9q deletions showed large partial chromosomal losses of similar size among all tested tumors. From this, we can speculate the possibility of a derivative (3;9) resulting from an unbalanced translocation as the common mechanism of chromosomal alteration in ELST.

The molecular similarities between ccRCC and ELST may make the diagnosis of ELST even more challenging and we emphasize the importance of integrating the clinical presentation with the radiological features of this tumor. Certainly, the presence of 3p loss in both tumors suggests a similar mechanistic origin of tumorigenesis between the two entities that warrants further investigation. Furthermore, ELST may benefit from therapies targeting the same molecular pathways that lead to RCC development including HIF-1 and its targets such as VEGF, CA-9, and platelet derived growth factor (PDGF) [24, 29].

While this study is limited by small sample size due to the rarity of ELST, we believe the novelty of our findings and the implications for avoiding diagnostic pitfalls lend its strength. Additionally, the opportunity to better understand the pathogenesis of such a rare neoplasm as ELST make this a unique and meaningful investigation. Other potential limitations of the study include the small amount of tissue available for testing in some cases, the age of some samples impairing the quality of DNA, and the unavailability of tissue for more extensive molecular testing in several cases.

Although ELST are uncommon tumors, generally associated with VHL disease, the majority of cases in our cohort had no history of VHL disease or other associated neoplasms. While ELST may be the initial presentation of the disease, it is more likely that these are sporadic cases of ELST. As such, our findings may be generalizable to both sporadic and syndromic ELST cases. Furthermore, loss of 3p has been seen in a number of other human cancers and our findings of 3p loss in ELST further support the presence of one or more tumor suppressor genes including *VHL* in this region of the chromosome.

## Conclusion

In conclusion, our findings dispel the previously reported misconception that ESLT are negative for expression of PAX-2, PAX-8, and CA-9. Likewise, copy number profiling will not help differentiate ELST from metastatic ccRCC. Based on our panel of immunohistochemical stains, CD10 and RCC may prove more useful in discriminating between the two entities. These noteworthy findings have important implications for the diagnosis, study, and possibly treatment of ELST and further investigation into the molecular pathways involved in tumorigenesis is warranted.

## Additional file

Additional file 1: Pathway and protein interactions in endolymphatic sac tumor. (PDF 52855 kb)

## Abbreviations

CA-9: Carbonic anhydrase 9
ccRCC: clear cell renal cell carcinoma
CNS: Central nervous system
CT: Computed tomography
ELST: Endolymphatic sac tumor
EMA: Epithelial membrane antigen
FFPE: Formalin-fixed paraffin embedded
GFAP: Glial fibrillary acidic protein
H&E: Hematoxylin and eosin
MRI: Magnetic resonance imaging
NSE: Neuron specific enolase
PAX: Paired box
PDGF: Platelet derived growth factor
RCC: Renal cell carcinoma
SNP: Single nucleotide polymorphism
VEGF: Vascular endothelial growth factor
VHL: Von-Hippel-Lindau
