# Supplementary material for: Expression of renal cell markers and detection of 3p loss links endolymphatic sac tumor to renal cell carcinoma and warrants careful evaluation to avoid diagnostic pitfalls
Source: Acta Neuropathol Commun. 2018 Oct 19;6:107. doi: 10.1186/s40478-018-0607-0 (PMC6194746; doi:10.1186/s40478-018-0607-0)
Supplement: Supplementary file 1 — Pathway and protein interactions in endolymphatic sac tumor. (PDF 52855 kb) [file 40478_2018_607_MOESM1_ESM.pdf]

Path Designer New My Pathway 2

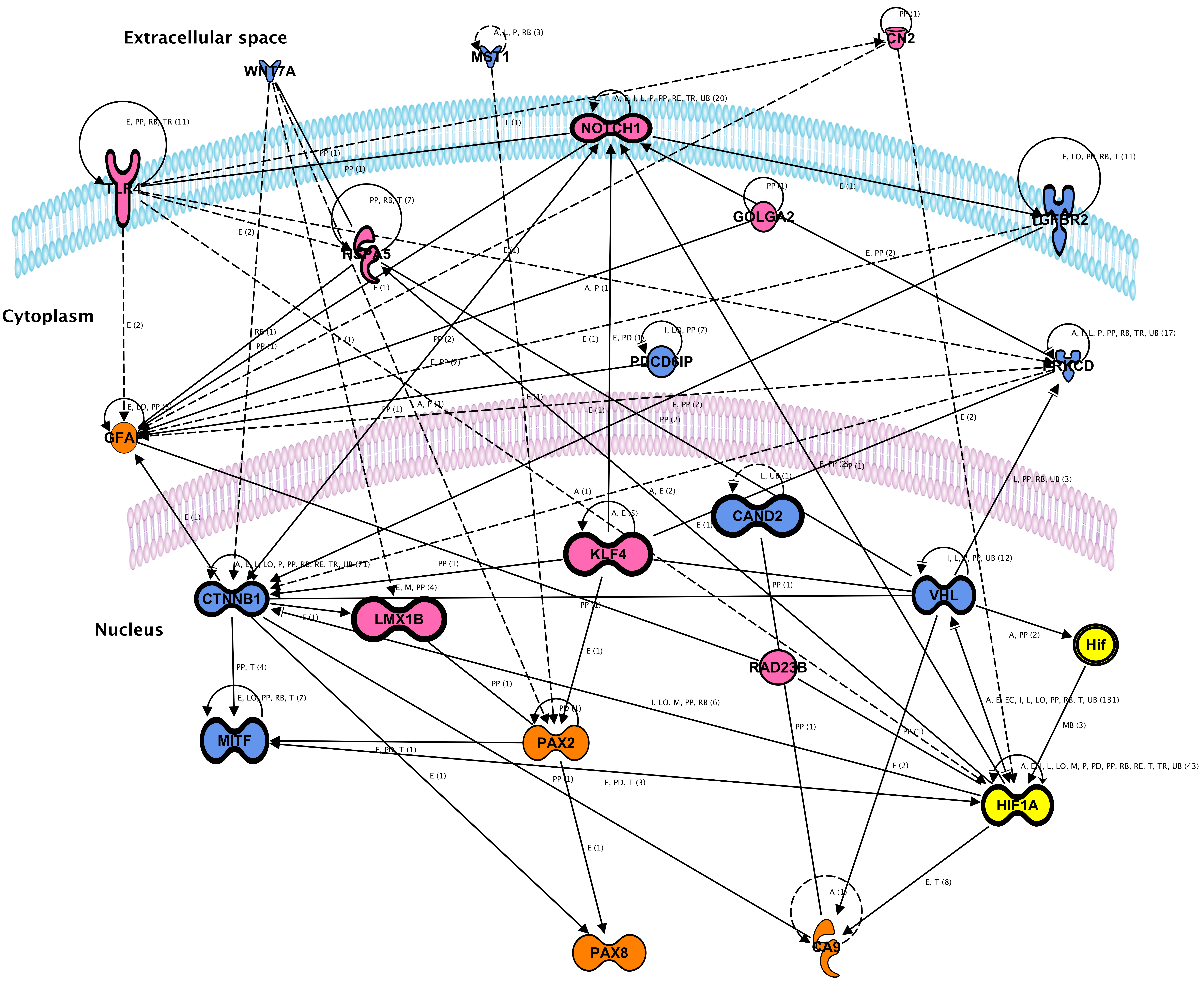

© 2000-2018 QIAGEN. All rights reserved.

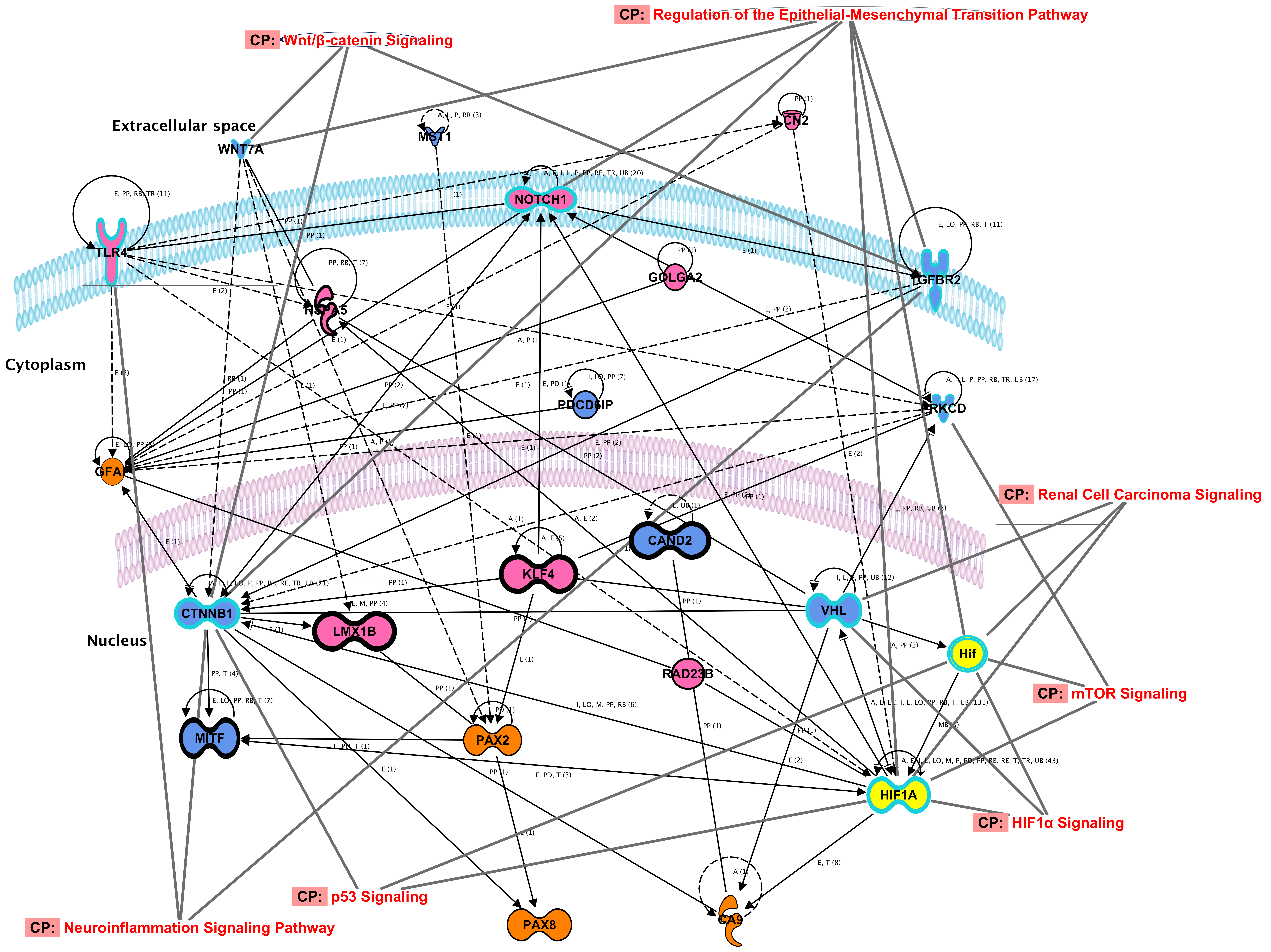

© 2000-2018 QIAGEN. All rights reserved.

Legend

Complex/group/other

Cytokine/growth factor

Enzyme

Transmembrane receptor

Transporter

Transcription regulator

Kinase

Blue: gene on 3p
Pink: gene on 9q

A — B: Chemical-chemical interactions, chemical-protein interactions, correlation, protein-protein interactions, RNA-RNA interactions: non targeting interactions

A → B: Activation, causation, expression, localization, membership, modification, molecular cleavage, phosphorylation, protein-DNA interactions, protein-RNA interactions, regulation of binding, transcription

A —| B: Inhibition, ubiquination

A —|→ B: Inhibits and acts on

A —> B: Reaction

Direct interaction

Indirect interaction

Relationship Labels

A	Activation
B	Binding
C	Causation/Leads to
CO	Correlation
CC	Chemical-Chemical interaction
CP	Chemical-Protein interaction
E	Expression (includes metabolism/ synthesis for chemicals)
EC	Enzyme Catalysis
I	Inhibition
L	Molecular Cleavage (includes degradation for Chemicals)
LO	Localization
M	Biochemical Modification
miT	microRNA Targeting
MB	Group/complex Membership
nTRR	Non-Targeting RNA-RNA Interaction
P	Phosphorylation/Dephosphorylation
PD	Protein-DNA binding
PP	Protein-Protein binding
PR	Protein-RNA binding
PY	Processing Yields
RB	Regulation of Binding
RE	Reaction
RR	RNA-RNA Binding
T	Transcription
TR	Translocation
UB	Ubiquitination
